# Supplementary material for: Molecular crowding effects on protein stability in a bacterial proteome
Source: Sci Rep. 2026 Jan 21;16:5908. doi: 10.1038/s41598-026-35990-9 (PMC12894976; doi:10.1038/s41598-026-35990-9)
Supplement: Supplementary file 2 — Supplementary Material 2 [file 41598_2026_35990_MOESM2_ESM.zip › AllTheTPPData/F70vF400/Panobinostat_Vignette/Melting_Curves/meltCurve_Q0K8S3.pdf]

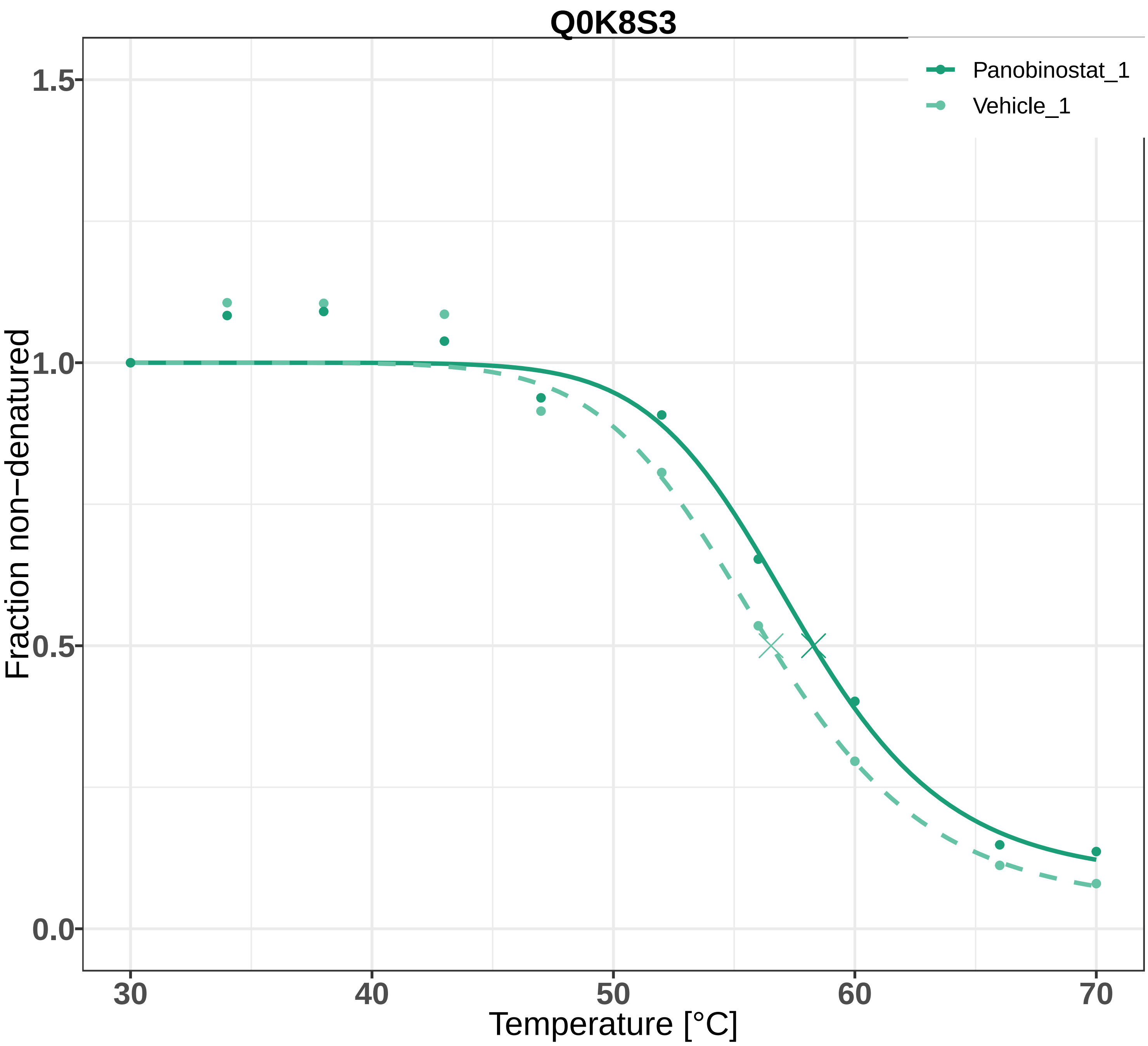

	meltPoint	slope	plateau	R2
**Panobinostat_1**	**58.29**	**−0.073**	**0.09**	**0.98**
**Vehicle_1**	**56.53**	**−0.07**	**0.04**	**0.98**
